# Prediction of Hepatocellular Carcinoma Prognosis and Immunotherapy Response Using Mitochondrial Dysregulation Features

**DOI:** 10.1111/jcmm.70389

**Published:** 2025-02-05

**Authors:** Jia Yu, Shengli Wu, Jinglong Gong, Wenbing Deng, Zhongsheng Xiao, LiangLiang Wu, Hong Long

**Affiliations:** ^1^ Department of Gastrointestinal Surgery, The First Affiliated Hospital, Hengyang Medical School University of South China Hengyang Hunan China; ^2^ Department of Hepatobiliary Surgery The First Affiliated Hospital of Xi'an Jiaotong University Xi'an Shaanxi China; ^3^ Department of Hepatobiliary Surgery, Hunan Provincial People's Hospital The First Affiliated Hospital of Hunan Normal University Changsha Hunan China; ^4^ Department of Gastroenterology, The First Affiliated Hospital, Hengyang Medical School University of South China Hengyang Hunan China; ^5^ Faculty of Data Science City University of Macau Macau SAR China

**Keywords:** immune infiltration, liver neoplasms, machine learning, mitochondria, precision medicine, prognosis, tumour biomarkers

## Abstract

Hepatocellular carcinoma (HCC) is a major contributor to cancer‐related deaths globally. Although there have been improvements in identifying treating the disease, patient outcomes are still unfavourable because of the significant variation in HCC. Mitochondrial‐related genes (MRGs) are crucial in tumour metabolism, cell death and immune response, emerging as potential therapeutic targets. We analysed 2030 MRGs using TCGA, GEO and HCCDB18 databases. Differentially expressed genes were identified using edgeR and limma, and enrichment analysis was performed via the clusterProfiler package. A prognostic model was built using machine learning algorithms and evaluated using LOOCV. Immune infiltration was assessed with CIBERSORT, EPIC, MCPCounter and TIMER algorithms, and drug sensitivity was analysed using the CTRP and PRISM datasets. MRG expression levels are significantly associated with worse outcomes in HCC patients outperformed conventional clinical indicators in immune response revealed that individuals at high risk exhibited weaker immune responses, characterised by reduced immune scores, and elevated levels of CD8+ T cells and macrophages. Notably, high‐risk patients also displayed heightened susceptibility to chemotherapy agents such as paclitaxel and irinotecan. Abnormal MRG expression serves as a significant biomarker for HCC prognosis. The developed model accurately predicts disease progression and can guide personalised treatment, especially for immune and chemotherapeutic therapies. Further validation with broader clinical samples is needed.

## 1 Introduction

Despite some progress in the early diagnosis and treatment of hepatocellular carcinoma (HCC), its morbidity and mortality rates remain high. The long‐term survival rate of patients with HCC is still not optimistic, and it is still one of the leading causes of cancer‐related deaths worldwide, posing a serious challenge to public health [1, 2, 3, 4]. The complexity of treating HCC lies in its multifactorial aetiology and heterogeneity. The heterogeneity of HCC often prevents a single treatment method from achieving the desired effect [3, 5]. Therefore, it is particularly important to seek more personalised treatment strategies. Mitochondria, the cell's powerhouse, are crucial in the onset and advancement of numerous diseases. Notably, dysfunctional mitochondria are strongly linked to the formation of tumours, influencing vital processes such as tumour growth, cell survival and communication pathways [6, 7, 8]. Therefore, targeted therapy strategies targeting mitochondrial‐related genes (MRGs) can effectively inhibit tumour growth by regulating energy production and metabolic pathways in tumour cells, while also reducing the tumour's resistance to traditional chemotherapy, demonstrating their great potential for clinical application [6, 9] and providing a new direction for precision medicine for HCC. Studies have shown that the regulation of mitochondrial function can not only affect the energy metabolism of tumour cells but also regulate the redox balance and immune response in the tumour microenvironment [10, 11]. Authoritative studies have found that mitochondrial transport proteins promote HCC progression by inhibiting iron death and anti‐tumour immunity [12]. There are also new materials specifically targeting mitochondria in chemo‐photodynamic nanoplatforms that are capable of disrupting the integrity of mitochondria, thereby inducing apoptosis by enhancing the accumulation of reactive oxygen species, which can help to improve the therapeutic efficacy of HCC [13]. Again, targeted drugs that target mitochondria have shown good imaging capabilities and therapeutic effects. Mitochondrial targeted therapy can enhance the effects of chemotherapy and radiotherapy by promoting apoptosis of tumour cells and reducing their ability to recover [14, 15].

Besides, abnormal expression of MRGs is also considered a potential biomarker for predicting HCC progression and treatment response [16, 17]. Monitoring these genetic changes in real time can help doctors better adjust treatment plans and implement personalised treatment strategies to achieve the best possible treatment results. Therefore, combining targeted mitochondrial therapy with traditional methods may be a key strategy for overcoming the challenges of treating HCC [15, 18]. This study explored the potential of MRGs in precision medicine by comprehensively analysing the expression patterns and functional abnormalities of MRGs in HCC patients. A predictive model using MRGs was created to investigate the impact of these genes on the metabolism of tumour cells and their immune responses. The key role of MRGs in the tumour microenvironment and their potential regulatory mechanisms were revealed.

## 2 Methods

### 2.1 Data Download and Preparation

The overall conceptual framework of this study is illustrated in Figure S1. We obtained transcriptome data for liver cancer (TCGA‐LIHC) and pan‐cancer, including survival data, from the UCSC online repository. To verify the generality of the research results, we also downloaded the GSE116174 dataset from the GEO database. In addition, we used the data in the HCCDB18 database for further verification at the transcriptome level regarding which datasets have larger sample sizes and more complete demographics with a view to improving the generalisability of the study results. Besides, 2030 MRGs were obtained from previous studies [19].

### 2.2 Differential Analysis

The data were analysed for differences using the edgeR and limma packages. The criteria for differentially expressed genes (DEGs) were set as |LogFC| < 0.8 and *p*‐value < 0.05. A Venn diagram was employed to highlight the MRGs that were commonly differentially expressed.

### 2.3 Functional Enrichment Analysis

Genes with different levels of expression were analysed to determine their functional enrichment by using Gene Ontology, Kyoto Encyclopedia of Genes and Genomes (KEGG) and Reactome. ClusterProfiler was used for GO analysis. This included biological processes (BPs), cellular component categories (CCs) and molecular functions (MFs). The same package was used to conduct Reactome pathway analysis. The FDR for enrichment was set to 0.05.

### 2.4 Prognostic Model Construction

We constructed the prognostic model using key machine learning algorithms that significantly enhanced its performance, including Random Survival Forest (RSF), Elastic Net, Lasso Regression and Ridge Regression. The models were trained using Leave‐One‐Out Cross‐Validation (LOOCV), with mitochondrial gene‐expression data sourced from TCGA's LIHC dataset. The AUC value of each model and Harrell's concordance (C‐index) were used to evaluate its predictive capability.

### 2.5 Prognostic Analysis

We used the bootstrap function to generate 1000 samples. Then we computed Cox coefficients and filtered out genes with a *p*‐value less than 0.05. The forest plot shows the results of Cox regression. We used the survival package with the prognostic models to determine the optimal thresholds for the mitochondrial gene scores (MRPs), which allowed us to stratify the samples into low‐risk and high‐risk categories. Kaplan–Meier curves were then created to assess the difference in survival between these two groups. We also conducted univariate Cox and multivariate Cox analyses to examine the impact of clinical characteristics, mitochondrial gene computations and survival package.

### 2.6 Immunorelated Analysis

The ESTIMATE algorithm calculated the immune score, stromal scores and tumour purity for tumour samples using the IOBR package. The levels of immune cells' infiltration were compared using four different algorithms—CIBERSORT, EPIC, MCPCounter and TIMER. The predictive power of the immunotherapy efficacy model was evaluated by using the IMvigor210 data. Gene Set Enrichment Analysis was used to examine gene expression, enrichment and various functional pathways.

### 2.7 Mutational Analysis

Maftools was used to perform the mutational analysis. This analysed the mutation frequencies of genes, the types of mutations and the distribution of frequently mutated gene. The package also analysed mutations of genes within key pathways as well as gain and loss in gene copy number variations (CNVs).

### 2.8 Drug Sensitivity Analysis

The study used the Cancer Therapeutics Response Portal v2.0 and PRposing datasets from the Broad Institute for drug sensitivity analyses. The researchers used a linear regression to calculate the sensitivity of different risk groups for each drug by analysing gene expression data from cancer cell lines. AUC values that are lower indicate a higher sensitivity to drugs by the cells. The researchers also used the Spearman coefficient to assess the relationship between drug sensitivity and the model score.

## 3 Results

### 3.1 Differentially Expressed Gene Screening and Enrichment Analyses Revealed Key Genes Associated With the Prognosis for HCC

**FIGURE 1:**
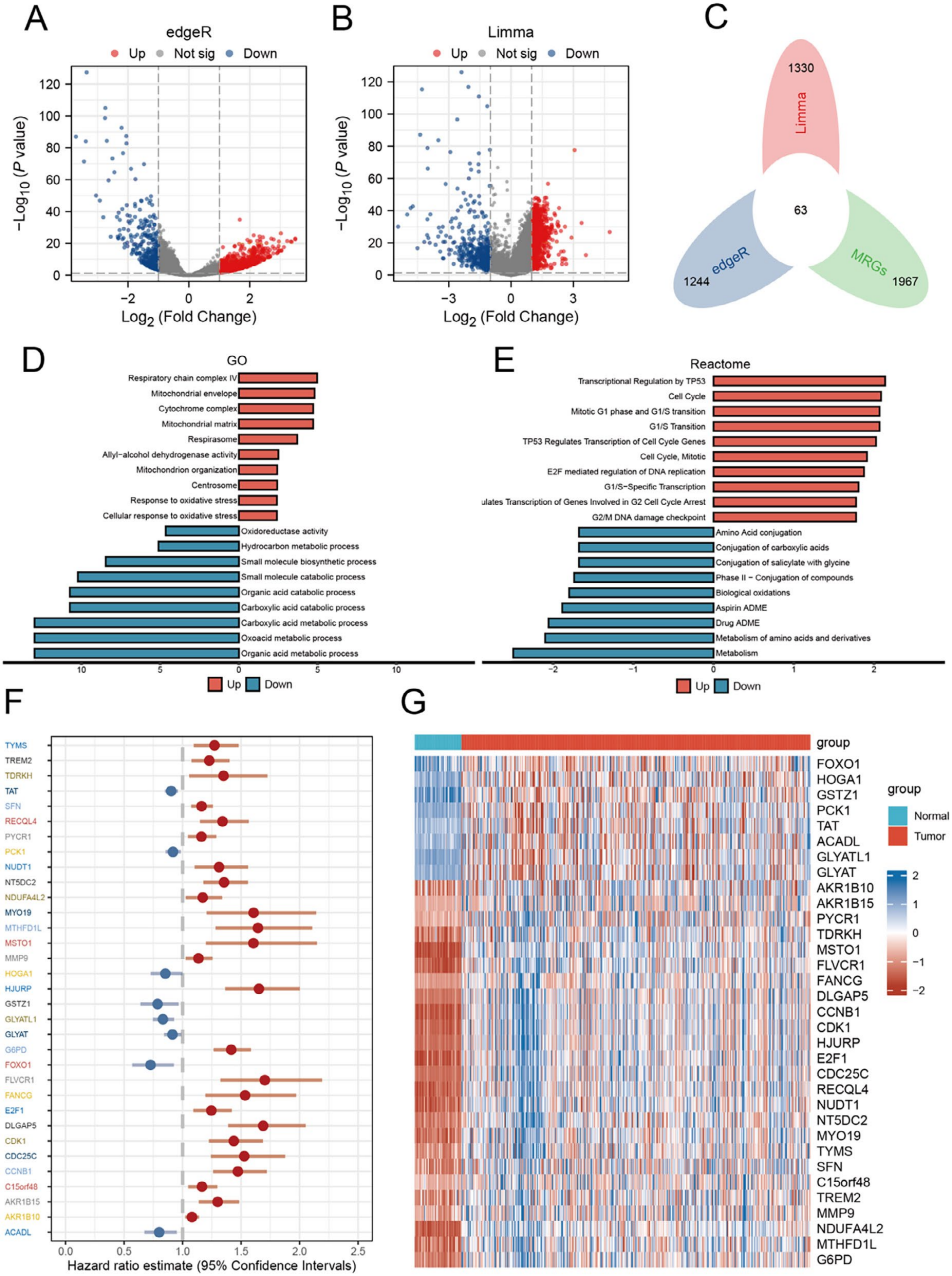
Testing and functional enrichment evaluation of differentially expressed genetics (DEGs) and expression patterns of essential prognostic genetics. (A) Volcano stories of the evaluated DEGs using lawn edger and (B) limma. (C) Venn layout showing the junction of the DEGs evaluated making use of lawn edger and limma with the mitochondrial‐related gene (MRG) collection, determining an overall of 63 crucial differentially expressed genes. (D) Enrichment results of the 63 genes in gene ontology (GO) evaluation. (E) Reactome path evaluation revealing the dramatically enriched pathways of the 63 genetics. (F) Woodland story showing the results of the Cox regression analysis of the 33 vital differentially shared genetics significantly related to the diagnosis of HCC individuals. (G) Heat map showing the expression patterns of the 33 essential prognosis genes in normal and tumour examples.

The edge identified 2674 DEGs (Figure 1A), whereas the limma found 1992 DEGs (Figure 1B). Both algorithms found that 63 of these genes were MRGs, and both identified them as differentially expressed (Figure 1C). These genes were found to be involved in processes relating to the respiratory chain complex IV, mitochondrial envelope, organic acid metabolism process and carbonyl acids metabolic process (Figure 1D). The Reactome Analysis revealed significant enrichment in DEGs for pathways like TP53 transcriptional control, cell cycle and metabolism (Figure 1E). Thirty‐three of these genes were associated with the prognosis of HCC patients (Figure 1F). A heat map was created to visualise the expression of 33 genes associated with HCC prognosis (Figure 1G).

### 3.2 Prognostic Model Building and Construction and Efficiency Assessment

**FIGURE 2:**
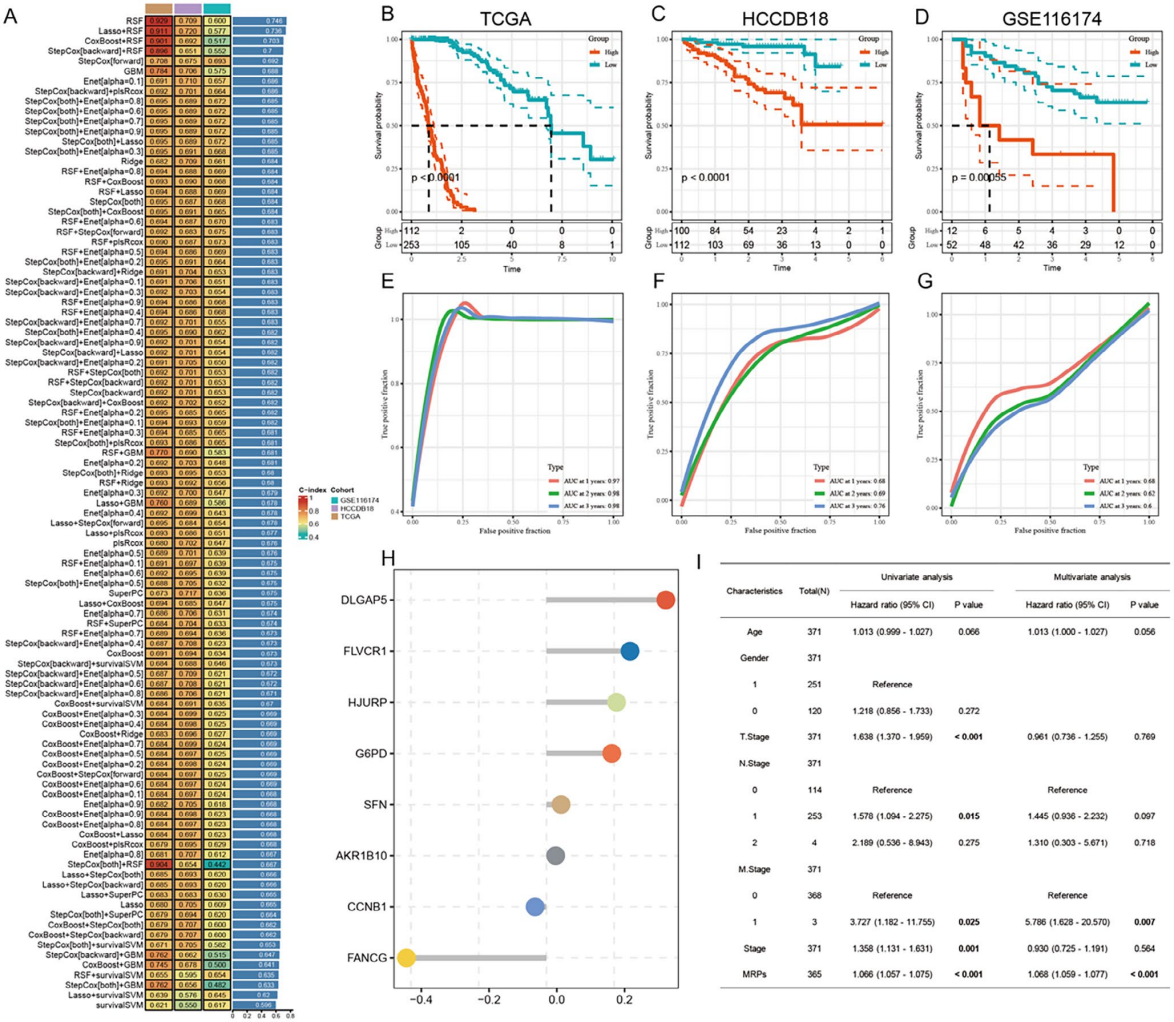
Prognostic model construction and validation, and evaluation of their predictive performance in different datasets. (A) Heat map showing the performance of various prognostic models constructed based on a combination of 101 machine learning algorithms. (B–D) Kaplan–Meier survival curve analysis based on the TCGA, HCCDB18 and GSE116174 datasets to demonstrate the significant survival difference between the high‐risk group and the low‐risk group. (E–G) The receiver operating characteristic (ROC) curve analysis of MRPs at 1, 3 and 5 years shows the predictive performance on different datasets. (H) The forest plot shows the results of the multivariate Cox regression analysis. (I) The results of univariate and multivariate Cox regression analyses show the impact of various clinical characteristics and mitochondrial‐related gene (MRG) scores on patient survival.

Referring to previous studies and further constructing the prognostic model, the RSF model presented the highest superiority among 101 machine learning algorithm combinations, exhibiting the highest C‐index on both the training and validation sets (Figure 2A). Figure 2B–D shows the Kaplan–Meier survival contours based upon the TCGA, HCCDB18 and GSE116174 datasets. The Kaplan–Meier curves showed significant differences in survival between high‐risk and low‐risk teams, with a higher‐risk group showing a worse survival diagnosis (*p* < 0.05). The model was highly predictive in all datasets with AUCs greater than 0.7 for 1‐, 3‐ and 5‐year prediction (Figure 2E–G). The forest plot is shown in Figure 2H, which shows the results of the multifactor Cox analysis. Important gene scores (such as DLGAP5 and others) are strongly associated with survival. Figure 2I shows the results of the univariate Cox analysis and the multivariate Cox analysis. It also shows the impact on patient survival of the different scientific information groups and MRGs. The multifactor analysis revealed that MRGs, as well as other medical features such as M‐staging, are independent prognostic factors. MRGs were the most significant.

### 3.3 Model Validation and Comparison

**FIGURE 3:**
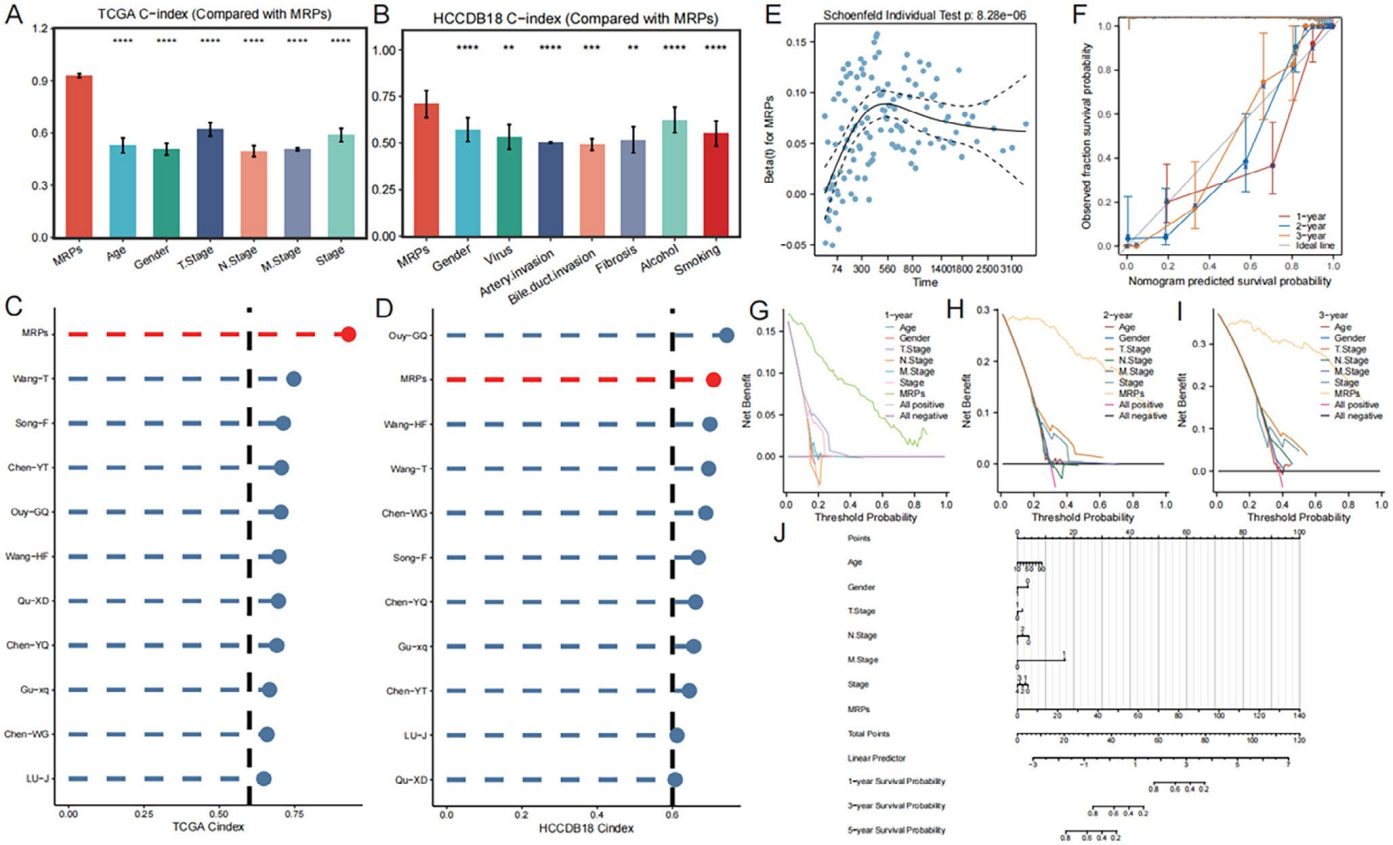
Validation of the prognostic model, and comparison and assessment of its potential application at different decision thresholds. (A, B) C‐index histograms on the TCGA and HCCDB18 datasets. (C, D) Comparison of the C‐index of MRPs with other key models on the TCGA and HCCDB18 datasets. (E) Scatter plot of prognostic proportional hazards. (F) The calibration curve demonstrates the degree of fit between the predicted survival probability of the model and the actual observed survival probability. (G–I) Decision curve analysis (DCA) evaluates the net benefit of the model at different decision thresholds at different time points. (J) Nomogram constructed based on a multifactor Cox regression model.

Figure 3A,B illustrates the C‐index histograms for our model on the TCGA dataset and the HCCDB18 data set. The model's high C‐index (both > 0.7) in both datasets indicates that it has good discriminative abilities. The C‐index of our model on the TCGA dataset and HCCDB18 shows a clear edge over other key models (Figure 3C,D). The scatter plot for the prognostic risk proportional shows that many of the points cluster around the diagonal. This indicates that the model accurately predicts the risk ratio, which is close to actual risk (Figure 3E). The model's predicted survival probability is very close to that of the observed survival probability (Figure 3F), indicating a good calibration. The net benefit from using the model for making decisions at different thresholds is greater than other strategies. This indicates the potential value of this model in clinical decision‐making (Figure 3G–I). On the basis of a multifactor Cox regression, the nomogram predicts individualised survival probabilities at 1 and 2 years (Figure 3J).

### 3.4 Comparison of the Potential Functions and Clinical Characteristics of Different Risk Groups

**FIGURE 4:**
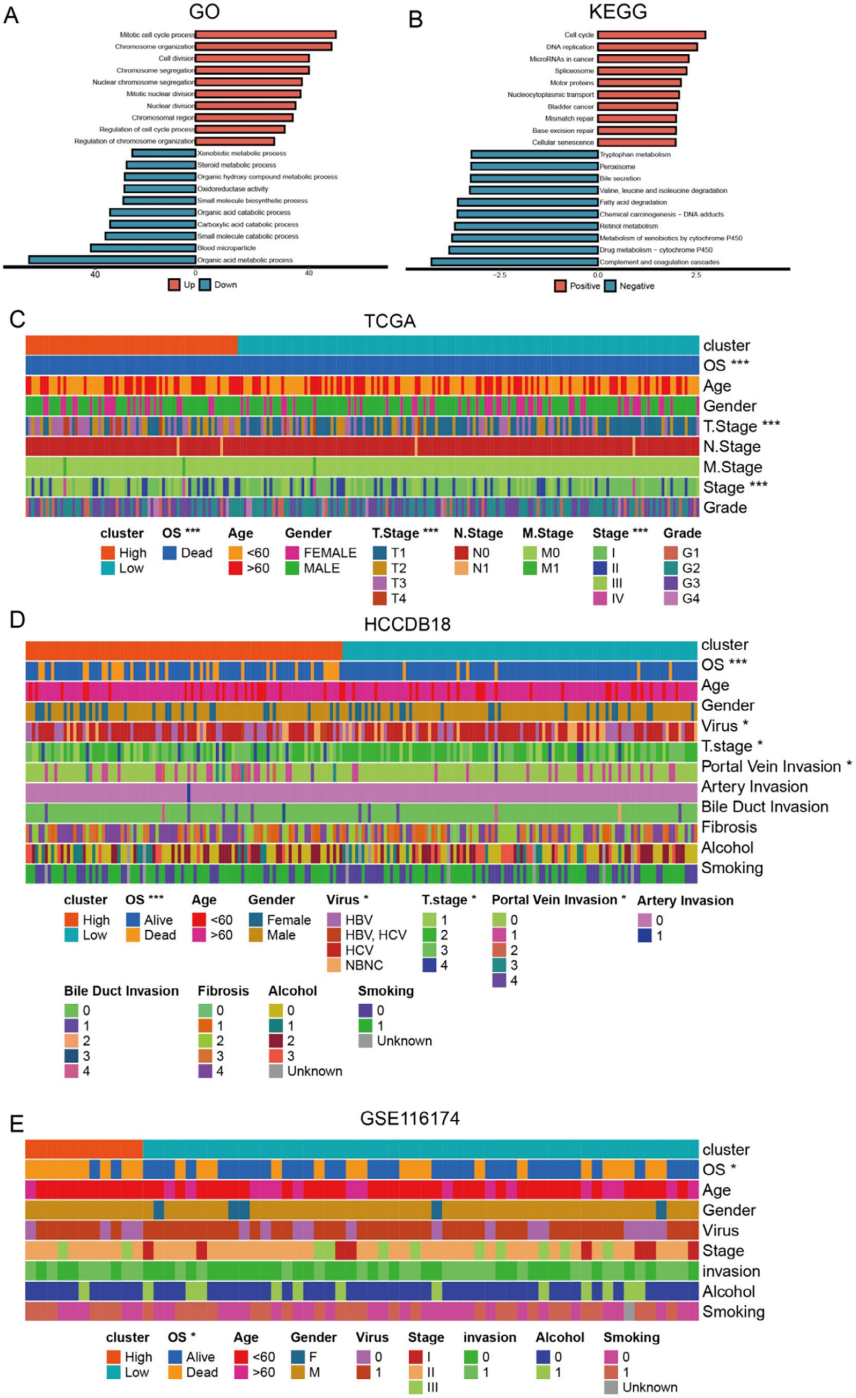
Comparison of potential functional enrichment and clinical characteristics of different risk groups. Enrichment analysis results of DEGs in different risk groups in (A) GO and (B) KEGG pathways. (C) Comparison of clinical characteristics of high‐risk and low‐risk groups in the TCGA, (D) HCCDB18 and (E) GSE116174 datasets.

The DEGs of different risk groups are mainly enriched for BPs like mitotic cell cycles, cell division and organic acid metabolism processes (Figure 4A). KEGG analysis revealed that these genes are significantly enriched for key pathways, such as cell cycle and replication of DNA (Figure 4B), closely linked to the progression and response to treatment of hepatocellular cancer. In comparing the distribution of clinical data between high‐risk and lower‐risk groups, there were significant differences in OS, T‐staging and staging between high‐risk and low‐risk groups. This was true for the TCGA dataset (Figure 4C) and the HCCDB18 dataset (Figure 4D). The GSE116174 dataset analysis showed that, although there was a similar overall trend, some clinical characteristics were less significant (Figure 4E).

### 3.5 Analysis of Immune Infiltrating Cells

**FIGURE 5:**
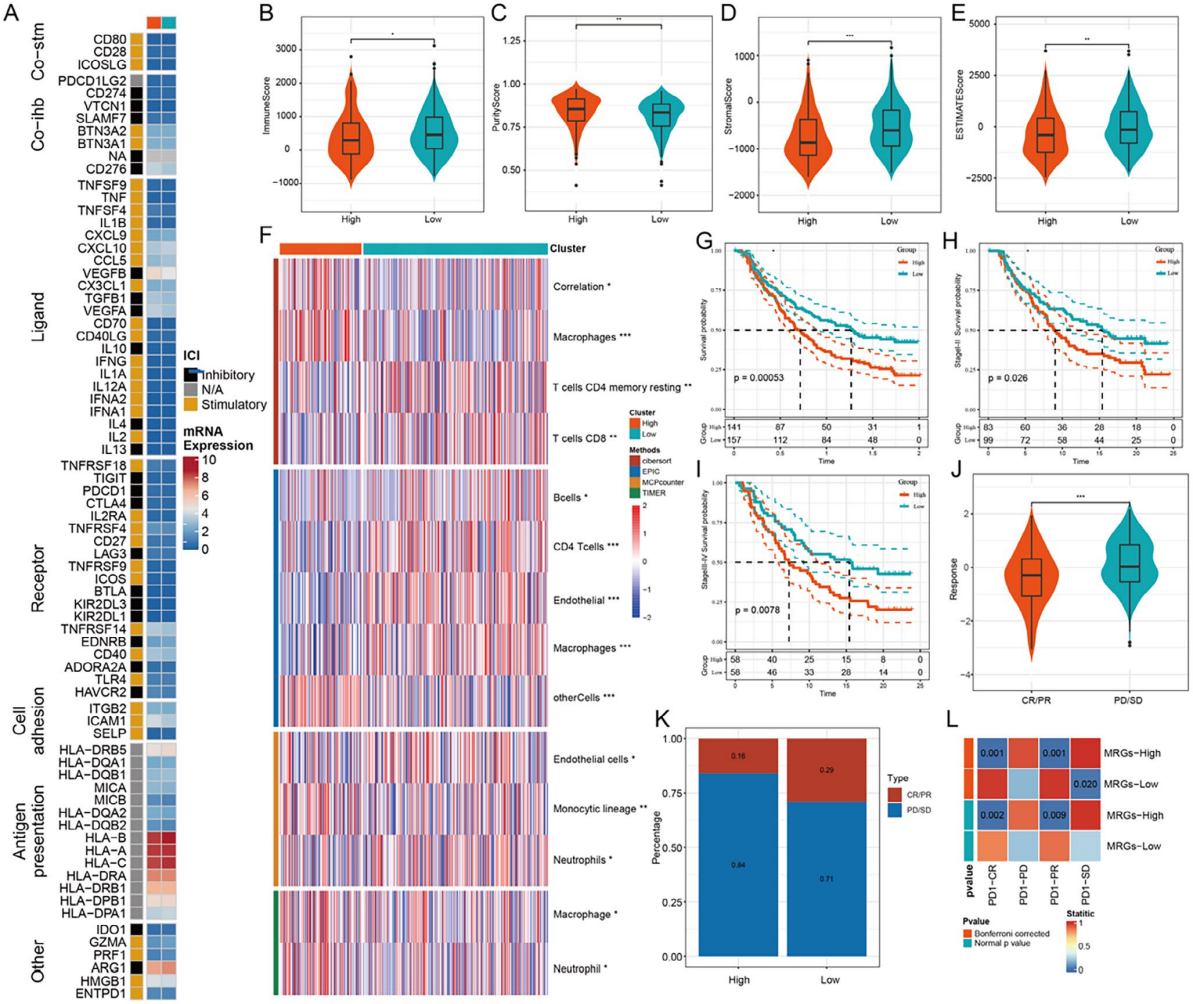
Immunological characteristics and immune infiltration analysis of patients in the high‐ and low‐risk groups. (A) Heat map of expression of immune‐related genes. (G) Survival rates of patients in the high‐ and low‐risk groups and of patients in stages I–II and (I) III–IV. (J) Immunotherapy remission rates and (K) efficacy ratios of patients in the high‐ and low‐risk groups. (L) Submap analysis showing the difference between patients in the high‐risk group and the low‐risk group in terms of immunotherapy responsiveness.

**FIGURE 6:**
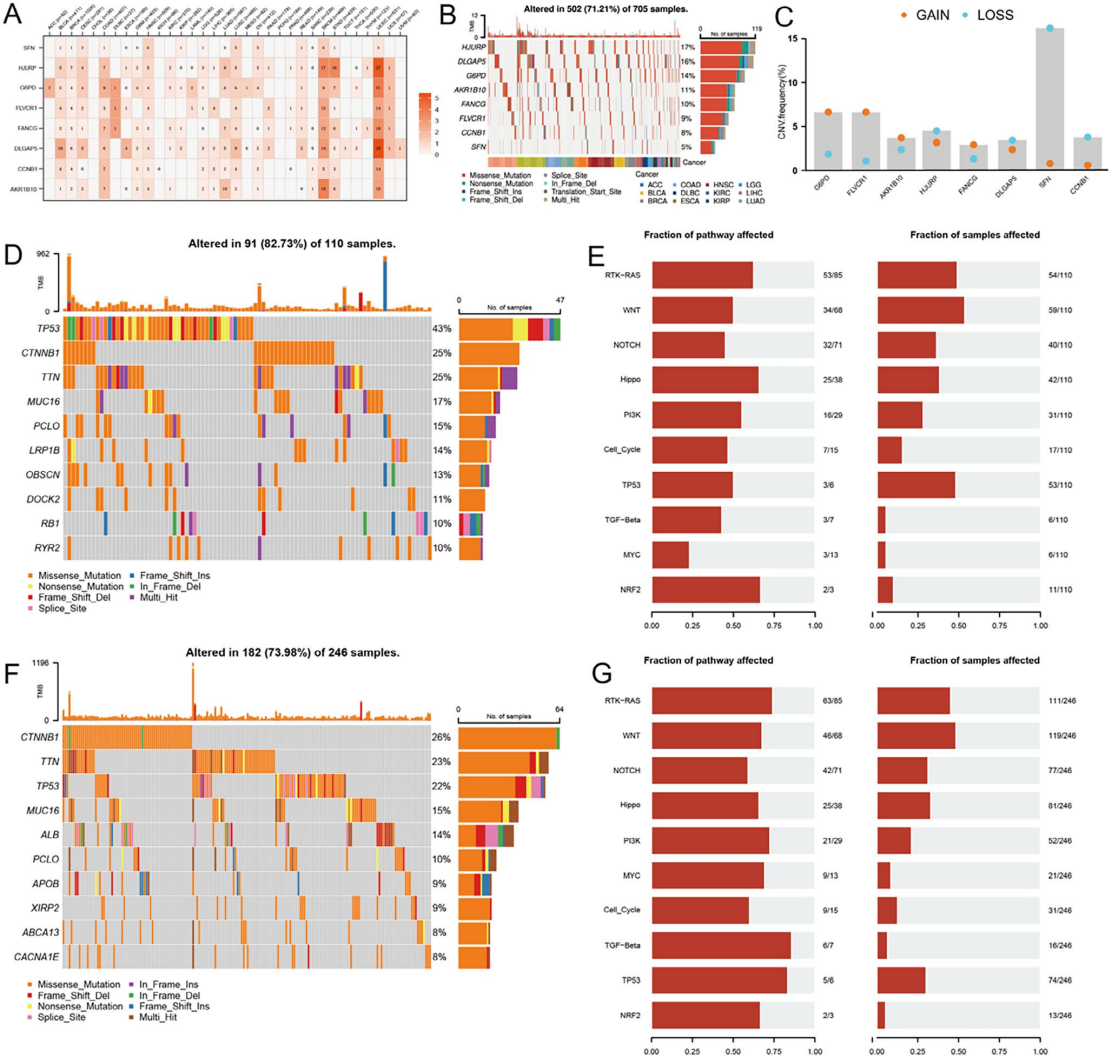
Mutation characteristics of key genes in MRPs in various cancers. (A) Expression correlation analysis and (B) gene mutation frequency analysis of key genes in various cancers. (C) Gene copy number variation (CNV) frequency, highlighting the high CNV loss frequency of the SFN gene in mutant samples. (D, F) Gene mutation status in high‐risk groups and low‐risk groups. (E, G) Pathway impact analysis.

Figure 5A shows that there are differences between high‐risk and low‐risk groups in terms of the expression of genes related to immune function. These include immunosuppressive and immune activating genes, as well as chemokine, cytokine and cytokine‐related genes. The high‐risk group had lower immune scores, and the stromal score was also lower (Figures 5B–E). The analysis using four algorithms for immune infiltration revealed significant differences between the high‐risk group and the lower‐risk groups, with the higher‐risk group showing greater levels of macrophage and CD8+ T cell infiltration (Figures 5F and 6F). In the IMvigor210 dataset, patients in the high‐risk group demonstrated a worse prognosis (Figure 5G). Even across clinical staging, the high‐risk group showed worse immunotherapy survival (Figure 5H,I), and patients in the high‐risk group had a significantly lower immunotherapy remission rate than those in the low‐risk group (Figure 5J,K). The submap analysis revealed that the high‐risk group showed a statistically significant difference in response to immunotherapy (Figure 5L).

### 3.6 Genetic Mutation Feature Analysis

Figure 6A illustrates the correlation between the expression of eight genes of MRPs and various cancers. It is evident that the expression patterns for these genes are different in each cancer type. Figure 6B shows the frequency of mutations in these genes. Out of 705 samples, there were 502 mutations (71.21%), with HJURP DLGAP5 G6PD being the most common. Figure 6C shows the CNVs for these genes. The SFN gene is particularly prominent with a CNV loss rate of 14%. Figure 6D,F illustrates the most common mutations found in high‐ and low‐risk groups. The highest mutation frequency was found in the high‐risk group for TP53, CTNNB1 and TP53. Figure 6E,G assesses the impact of pathways. It is shown that the pathways RTK‐RAS and WNT are affected significantly in the high‐risk groups, whereas the impact in the low‐risk group is low. These results show that there are differences between high‐risk and low‐risk groups in terms of gene mutations and signal pathways, which may indicate their role in prognosis.

### 3.7 Analysis of Drugs

**FIGURE 7:**
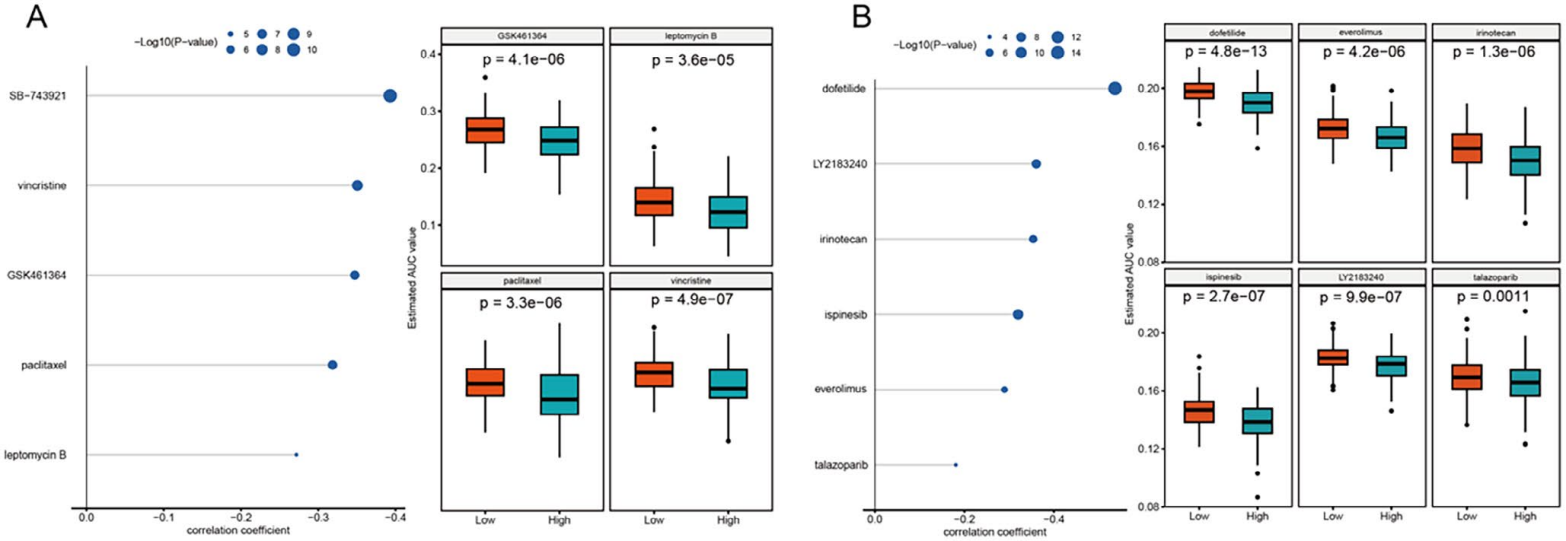
Antibiotic susceptibility analysis. (A) CTRP database analysis results. (B) PRISM database analysis results.

In the CTRP database (Figure 7A), SB‐743921 and vincristine showed significant negative correlations. This indicates that the increased activity of the drugs is associated with decreased drug sensitivity and that the patients in the high‐risk group are more susceptible to the drugs. The PRISM database results (Figure 7B) show that the patients who are at high risk have a higher sensitivity to certain drugs, such as everolimus, irinotecan and dofetilide. LY2183240 and ispinesib also show a similar pattern between risk groups. This highlights how differences in gene expression can affect a patient's response to treatment.

### 3.8 Analysis of Pathway Enrichment and Correlation Between Key Prognostic Gene and Immune Infiltration Characteristics

**FIGURE 8:**
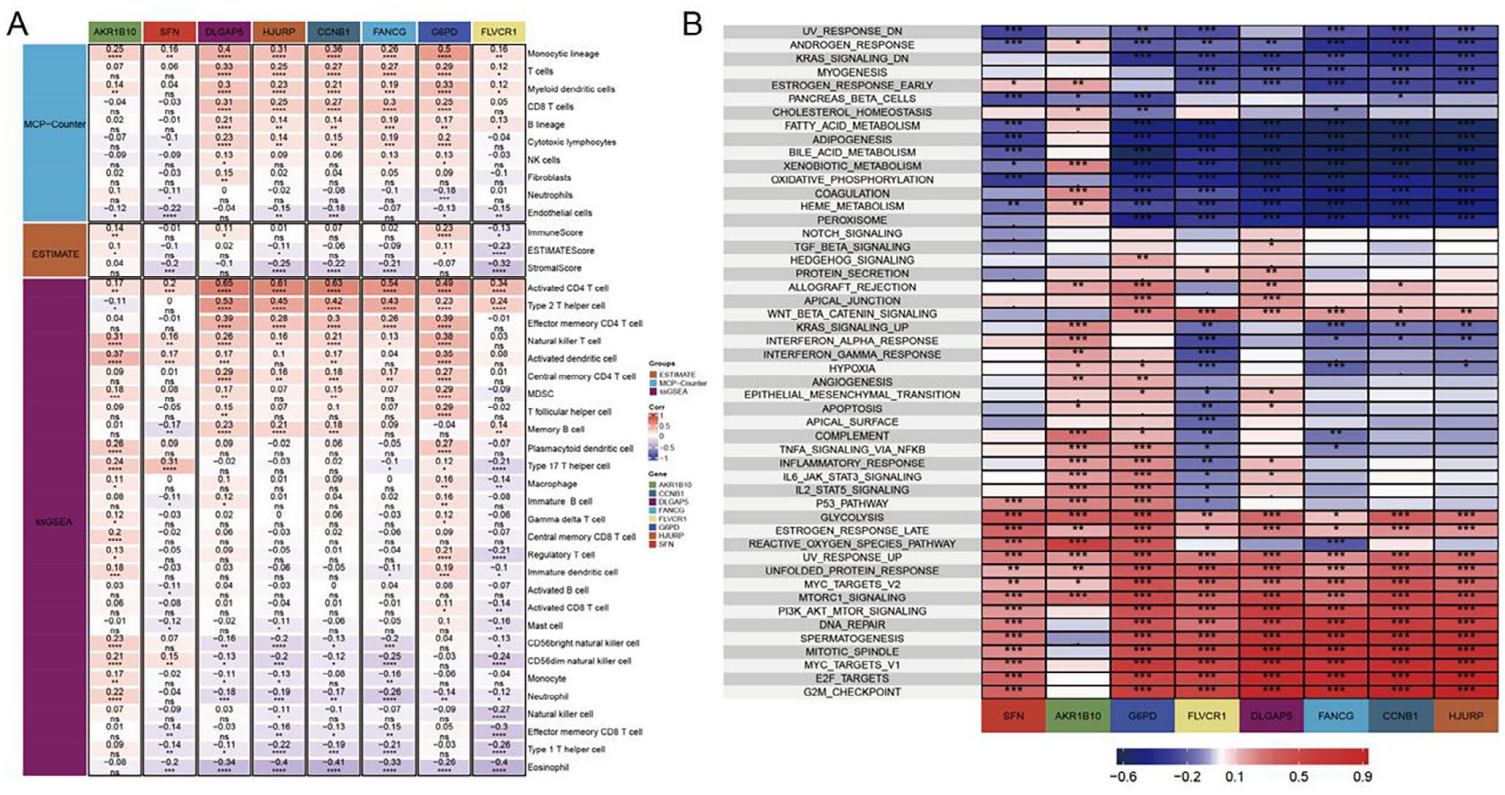
Correlation analysis of key prognostic genes and enrichment of immune infiltration features and pathways. (A) Correlation between selected key prognostic genes and immune cell infiltration features. (B) GSEA results based on key prognostic genes.

The results revealed that the high expression of some genes (such DLGAP5) is significantly associated with the high infiltration rate of immune cells such as CD8+ T cells (Figure 8A). Figure 8B displays the GSEA results using key prognostic gene. These genes were significantly overrepresented in pathways that are important for tumour biology such as the G2M_CHECKPOINT cell cycle and MTORC1 SIGNALLING cell cycle. This suggests that these genes could play a role in HCC through regulating biological functions.

### 3.9 Correlation Analysis of MRP Genes With Prognosis and Immune Microenvironment

**FIGURE 9:**
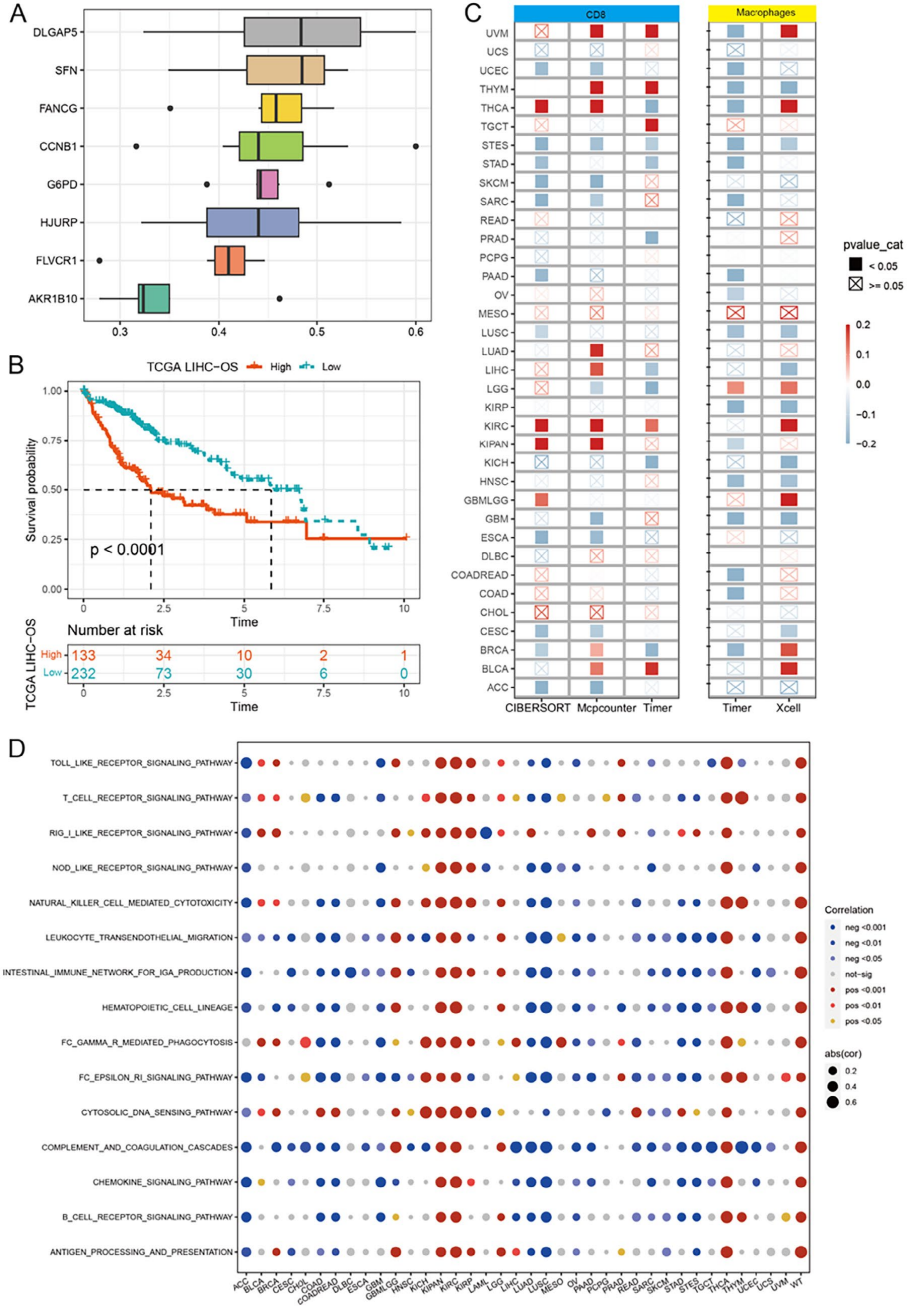
Cox regression analysis of the key prognostic gene DLGAP5 and its relationship with the immune microenvironment. (A) Cox regression analysis‐based analysis of the relationship between eight key genes in MRPs (such as DLGAP5 and SFN) and patient prognosis. The figure shows the hazard ratio (HR) of each gene and its 95% confidence interval. (B) Kaplan–Meier survival curves showing the relationship between DLGAP5 expression levels and overall survival (OS) of patients. The survival rate of patients in the high DLGAP5 expression group was significantly lower than that in the low expression group (*p* < 0.0001). (C) Correlation matrix of DLGAP5 expression levels with immune cell infiltration. The relationship between the infiltration density of CD8+ T cells and macrophages and DLGAP5 expression in different cancers was analysed using immune infiltration analysis tools such as CIBERSORT, MCPCounter and TIMER. (D) The results of GSEA based on DLGAP5 expression show a positive correlation between high DLGAP5 expression and multiple immune‐related pathways, indicating the potential role of DLGAP5 in regulating the tumour immune microenvironment.

Cox regression was done on eight genes that make up MRPs. The results revealed that certain genes, like DLGAP5 or SFN, had significant prognostic values (Figure 9A). The prognosis of patients in the group with high DLGAP5 levels was significantly worse (Figure 9B). DLGAP5 in cancer was also significantly associated with the density of macrophages and CD8+ T cells infiltrating into different tumours (Figure 9C). DLGAP5 was associated with an increase in immune‐related pathways, such as T cell receptor pathway and B cell receptor pathway (Figure 9D), This confirms the importance of these genes for hepatocellular cancer.

## 4 Discussion

With the wide application of precision medicine in cancer treatment, the key to HCC treatment lies in identifying reliable molecular markers and developing effective molecularly targeted therapy strategies to address its complex genetic characteristics and tumour heterogeneity, thereby improving treatment outcomes [19, 20]. Although the importance of mitochondria in cancer metabolism and immune regulation has been reported [11, 21, 22, 23], the specific mechanism of action of MRGs in the prognosis and immune microenvironment of HCC remains to be further investigated. In particular, how to integrate MRGs into clinical prediction models to guide personalised treatment decisions has not been fully explored. This study further explored the key regulatory role of these genes in tumour progression by digging deeper into the role of MRGs in HCC and provided new research perspectives for the development of personalised treatment strategies. This study first constructed prognostic model based on machine learning algorithms. The RSF model had the highest C‐index and the best predictive performance of 101 combinations. MRPs have been shown to accurately differentiate between low‐risk and high‐risk groups in the TCGA datasets. They also performed well in the 1‐, 3‐, and 5‐year prediction. The MRG score was found to be superior even to other clinical factors such as M‐staging in predicting the outcome of patients with HCC.

This study also revealed that DEGs from different risk groups are mainly enriched for biological pathways, such as mitotic cell cycles, cell divisions and metabolic processes. They were particularly enriched in pathways like the cell cycle and replication of DNA, which suggests that MRGs are important in HCC tumour growth and response to treatment. MRGs regulate the cell cycle, which affects the drug resistance and sensitivity of tumours. Studies have shown that mitochondrial dysfunction may promote tumour cell growth by regulating abnormal cell cycles. It could also exacerbate immune evasion or immunosuppression within the tumour microenvironment [24, 25]; this also confirms our results. There were also significant differences between the risk groups in terms of immune scores, tumour purity and stromal scores. The immune score, tumour purity and stromal scores also showed significant differences. Patients with high risk showed greater immune suppression and poorer responses to immunotherapy. Patients in the high‐risk category showed higher levels of immune suppression and had poorer immunotherapy responses. This is consistent with previous studies [26, 27] showing that dysregulation of mitochondrial function is closely associated with tumour immune escape and the immunosuppressive state of the tumour microenvironment.

The IMvigor210 dataset also showed that patients in high‐risk groups had lower immunotherapy efficacy and a significantly lower immunotherapy response rate than those in low‐risk groups. This suggests that MRGs could directly affect immune cells' function and the tumours immune escape mechanism through altering cytokines or chemokines expression in the tumour microenvironment. Similar studies [22, 28, 29, 30] have also found that by regulating these risk genomes, the immune characteristics of the tumour microenvironment can be significantly affected, further verifying the key role of MRGs in regulating the immune response. Furthermore, mutation analysis showed that the frequency of TP53 and CTNNB1 mutations was significantly higher in the high‐risk group, indicating that MRGs not only play a key role in tumour progression but also are closely related to genetic variation in tumours. TP53 mutations in HCC are common, particularly in hepatitis B cancers. They are associated with abnormal cell cycle regulation, which allows the cells to avoid apoptosis and increase the likelihood of metastasis and tumour growth [31, 32]. Chen et al. [33] stated that CTNNB1 mutations in HCC are a prognostic marker that is independent of immunotherapy. It is manifested by a decrease in the activation and increase in the immunosuppressive molecule in the immune environment. This affects the response to immune checkpoint inhibitor therapy. The potential of MRPs to treat HCC was further confirmed by a drug sensitivity analysis. Patients in the high‐risk group were more sensitive to various chemotherapeutics (like paclitaxel and irinotecan). The results suggest that drug sensitivity analyses based on MRGs can be used to guide precision treatment for HCC. Recent studies have demonstrated that ultrasound contrast agent‐coated nano‐paclitaxel can effectively suppress HCC cell proliferation and invasion by blocking them in their G2/M phase and inducing apoptosis [34]. Li et al. [23] also found in their study that targeted nanoliposomes were able to exhibit enhanced cytotoxicity and increased levels of cellular uptake of the drug compared to free paclitaxel. Liu et al. [35] found in their study that the combination of irinotecan and TDO inhibitor significantly enhanced the therapeutic efficacy against HCC. By inhibiting production of kynurenine and increasing DNA damage in cancer cells, this treatment improved tumour immune microenvironment while inducing apoptosis in HepG2 cancer cells—opening up new possibilities for precise liver cancer treatments.

Although this study provides new insights into the role of MRGs in HCC prognosis, there are also some limitations. First, most of its data came from public databases and was not obtained directly. Although the validation set demonstrates the robustness of the model, more large‐scale clinical data are needed in the future to fully validate its practical application value. Second, although our research has demonstrated the role of MRGs in controlling the immune microenvironment of HCC, their specific mechanism requires further verification experimentally. Furthermore, future research should incorporate patients from diverse races and regions into its model to ensure universality and reliability of its predictions.

Overall, this research demonstrated the role of these genes in HCC by creating a prognostic model based on MRGs, and offered new ideas for personalised treatment and targeted therapy of HCC. Future research may explore more specifically their specific mechanisms and clinical application value to provide more comprehensive precision medicine solutions in HCC.

## Author Contributions

**Jia Yu:** conceptualization (equal), data curation (equal), writing – original draft (equal). **Shengli Wu:** data curation (equal), formal analysis (equal), software (equal). **Jinglong Gong:** formal analysis (equal), software (equal). **Wenbing Deng:** investigation (equal), methodology (equal), validation (equal). **Zhongsheng Xiao:** investigation (equal), methodology (equal), validation (equal). **LiangLiang Wu:** investigation (equal), methodology (equal), validation (equal). **Hong Long:** conceptualization (equal), data curation (equal), funding acquisition (equal), supervision (equal), writing – review and editing (equal).

## Conflicts of Interest

The authors declare no conflicts of interest.

## Supporting information


**Figure S1.** Graphical abstract of mitochondrial dysregulation in hepatocellular carcinoma (HCC). This graphical abstract provides an overview of the study design and principal findings. It highlights how mitochondrial dysregulation features were identified, integrated and employed to predict the prognosis of HCC and the likelihood of immunotherapy response.

## Data Availability

The datasets generated and/or analyzed during the current study are available from the corresponding author upon reasonable request.
