# Supplementary figures and images for: Prediction of Hepatocellular Carcinoma Prognosis and Immunotherapy Response Using Mitochondrial Dysregulation Features
Source: J Cell Mol Med. 2025 Feb 5;29(3):e70389. doi: 10.1111/jcmm.70389 (PMC11798730; doi:10.1111/jcmm.70389)

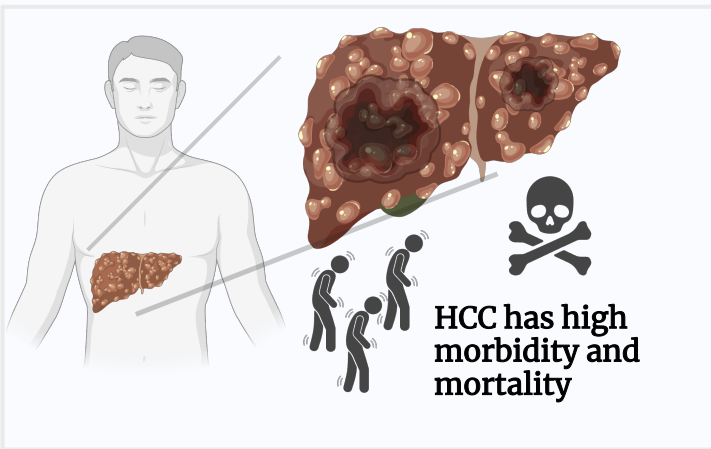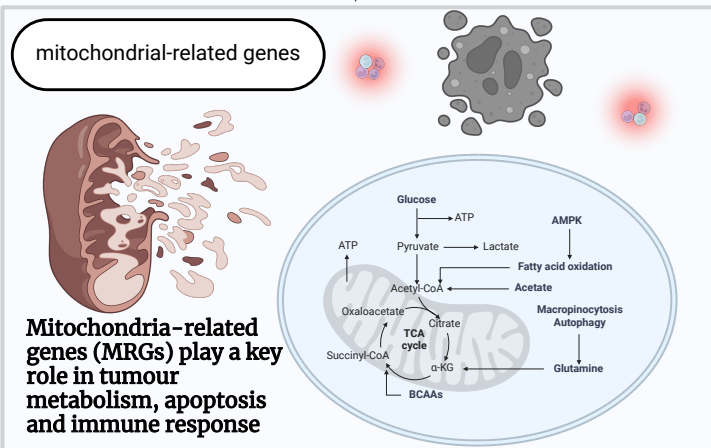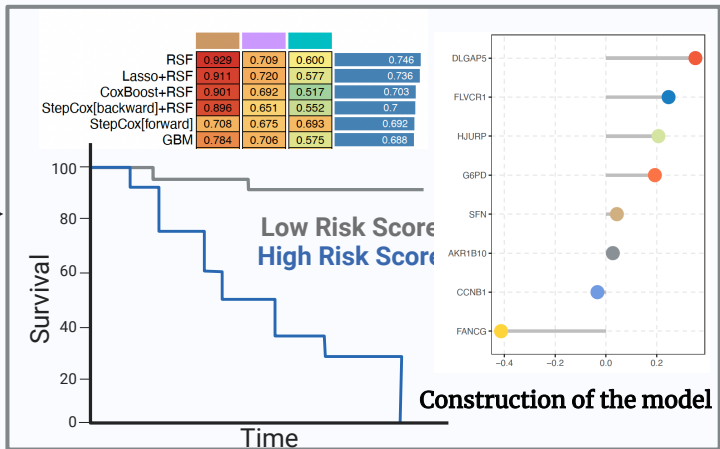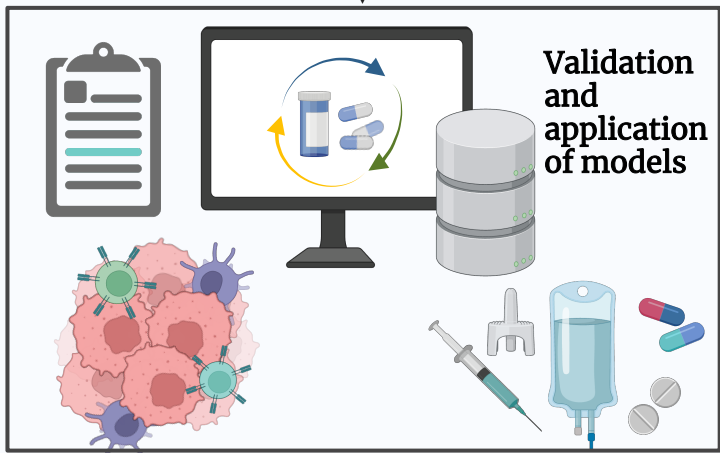

Supplement: Supplementary file 1 — Figure S1. Graphical abstract of mitochondrial dysregulation in hepatocellular carcinoma (HCC). This graphical abstract provides an overview of the study design and principal findings. It highlights how mitochondrial dysregulation features were identified, integrated and employed to predict the prognosis of HCC and the likelihood of immunotherapy response. [file JCMM-29-e70389-s001.pdf]
